# Trismus Nascentium

**Published:** 1846-10

**Authors:** 


### PART V.—ABSTRACTS.

### ARTICLE X.

### TRISMUS NASCENTIUM.

I. Marion Sims, M. D., of Montgomery, Alabama, in the April number of the American Journal of Medical Sciences, has published an interesting paper, in which some new views, particularly those relating to its cause and treatment, are advocated. Anything which promises to elucidate this imperfectly understood subject is especially valuable.

Dr. Sims insists that the true seat of the disease is in the spinal cord, that its morbid anatomy consists first in a congestion, and then in a rupture of the minute veins and capillaries of the medulla spinalis, producing extravasation, which view is supported by Goelis of Vienna, Thompson of Philadelphia, and M. Billard. The cause of this venous congestion etc., is a continued dorsal position of the infant, associated with an imperfect ossification of the cranial bones. Six cases are detailed, three occuring in his own, and three in the practice of Drs. Boswell and Jones, to whom the author had suggested his ideas of the pathology of this affection. In the first case the child had lain during the whole of its illness exactly in one position all the time; the weight of the head resting wholly on the os occipitis. The fontanelles were open and very large, particularly the anterior. The bones were loosely attached by their commissures, and the os occipitis was pushed in on the brain, being over-lapped for a quarter of an inch or more along the whole course of the lambdoidal suture, by the ossa parietalia.

In the second case the infant had lain on its back ever since its birth, thirteen days, resting principally on the mother's or grandmother's arm, and at the time of Dr. Boswell's visit was held by the latter with the occiput resting on her arm. The occipital bone was considerably pushed in, and overlapped by the parietal bones. In the third case the infant was lying on its back, with some coarse cloth doubled under its head; the overseer said he always found the child lying on its back. The os occipitis was pushed deeply under and overlapped by the parietal bones. In the fifth instance, the child was either lying in the objectionable position with its head on a pillow, or held on the lap with its occiput on the nurse's patella. The position of the cranial bones was as in the foregoing cases. In the sixth case, the position of the child and of the cranial bones were essentially the same; and in the fourth case the symptoms were produced by a tedious labor, by which, as the mistress of the mother said, the child's head was "mightily mashed."

The rationale of the pathology of this disease is as follows. When the fœtal cranium is not sufficiently ossified to regain its proper shape after parturition, by its own elasticity, or when the child is constantly retained in the positions before described, with the weight of its head resting on the occipital bone, especially when the head is supported by an unyielding substance, the whole cerebral mass will be displaced. The cerebellum will be compressed between the fossa cerebelli and the tentorium, and will be tilted forwards so as to produce great pressure on the whole tract of the medulla oblongata as it rests on the basilar process of the occipital bone. The circulation through the sinuses and veins of the brain is retarded, the posterior edge of the foramen magnum constricts that portion of the medulla-spinal veins which empty their blood into the inferior cerebella veins; whilst the medulla oblongata compresses that portion of the same veins which run forward over the anterior or lateral edges of the foramen magnum to empty into the pretrosal sinuses; thus producing congestion of, and extravasation from the medulla-spinal veins, which form a delicate tortuous network around the spinal cord, between the pia mater and the arachnoid.

In the only case in which an examination of the spinal cord was had, there was found a coagulum of blood occupying the spine its whole length, enveloping perfectly the medulla spinalis, thicker as it approached the brain. The spinal veins were full of black blood.

It may be objected to this pathology and its causes, that as the occipital bone is more or less displaced inwards and upwards in every labor, this disease should always or more frequently occur. To which, the following is an answer, as well as being a part of the explanation of the manner in which the extravasation occurs. Extravasation does not take place as soon as the constriction is made at the edges of the foramen magnum, because the medulla-spinal veins communicate with the great spinal lying exterior to the dura mater, and thus the blood is carried into the general circulation by the anastomoses with the vertebral, intercostal, azygos, lumbar, and sacral veins; and except in extreme cases, the compression in parturition is insufficient to produce the symptoms.

The case in which the child was born with its head "mightily mashed" is an illustration of the fact, that the compression at this period, when the bones of the head are much less ossified than usual, does occasionally produce trismus nascentium. In this instance it was present from the hour of its birth. But if the child remains for a considerable period in the position heretofore so often referred to, with the occipital bone forced in on the brain, the spinal veins all become congested. In this manner, the dorsi-spinal veins, which receive the blood from the muscles of the back, carry it horizontally forward between the vertebral arches into the meningo-rachidian, as they lie on the dura mater of the cord; they carry it forward into the general current of the circulation, emptying, by transverse branches, into the vertebral, superior intercostal, vena, azygos major and minor, and into the lumbar and sacral veins. This chain of venous trunks runs the whole length of the spinal column, and yet their contents are passed mostly horizontally forwards. There is no vis-a tergo to drive the blood horizontally forwards, or, as the child lies on its back, perpendicularly upwards, it almost ceases to flow, and the medulla-spinal ligated above, and dammed up on all sides, having no outlet for the blood brought down by the anterior and posterior spinal arteries, must necessarily yield, and pour out their contents into the dura mater.

The treatment consists in a rigidly careful prophylaxis. After the extravasation of blood the patient must almost necessarily perish. Of the six cases given two recovered, and in these as in the others, the treatment pursued was that of changing the position of the child, so that the weight of the head should fall on the parietal protuberance, on a soft pillow. When nursed of course the same precautions are to be observed. The application of blisters to the spine (in conjunction with proper posture) is suggested by Dr. Sims.

